# Prognostic value of HMGB1 overexpression in resectable gastric adenocarcinomas

**DOI:** 10.1186/1477-7819-8-52

**Published:** 2010-06-26

**Authors:** Guoqiang Bao, Qing Qiao, Huadong Zhao, Xianli He

**Affiliations:** 1 Department of general surgery, Tangdu Hospital, The Fourth Military Medical University, Xi'an 710038, China

## Abstract

**Introduction:**

HMGB1(High mobility group box 1), originally described as a nuclear protein, is now regarded as a multifunctional protein with a paradoxical dual effect in tumors. In the present study, HMGB1 overexpression and its correlation with the clinicopathologic characteristics and recurrence-free survival were evaluated in gastric adenocarcinomas.

**Methods:**

76 gastric adenocarcinomas surgically removed entered the study. The immunohistochemical staining was used to assess HMGB1 expression through tissue microarray procedure. The clinicopathologic characteristics of all patients were recorded, and the regular follow-up was made for all patients.

**Results:**

Almost all the gastric adenocarcinomas showed HMGB1 positive staining mainly in the nucleus, and the overexpression of HMGB1 was found in cancerous tissues with higher strong reactivity rate, compared with non-cancerous tissues (total expression score ≥ 9, 42.0% *vs*. 9.0%, *P *< 0.001). Survival analysis revealed that tumor stage negatively correlated with cancer-free survival (*P *= 0.022). Furthermore, HMGB1 overexpression positively associated with cancer-free survival of resectable gastric adenocarcinomas (*P *= 0.023).

**Conclusions:**

The overexpression of HMGB1 protein indicates that HMGB1 may play a role in the tumorigenesis of gastric adenocarcinomas. And the overexpression of HMGB1 may be a marker of good prognosis of gastric adenocarcinoma given curative resection combined with adjuvant chemotherapy.

## Introduction

Gastric cancer (GC) is the second most common cause of cancer-related death in the world. Many Asian countries, including China, have very high rates of GC. For patients in advanced stages, the five-year survival rate is only about 20 percent. There are many factors that limit the prognosis of the disease. High mobility group box l (HMGB1), a nuclear DNA-binding protein, originally described as a nuclear protein that binds to and modifies DNA, stabilizes the structure and function of chromatin and regulates gene transcription. It has been realized that HMGB1 can act either as a DNA binding protein or extracellularly as a cytokine-like danger signal, which is either actively secreted or passively released by necrotic cells[1]. Now HMGB1 is regarded as a central mediator of inflammation by acting as a cytokine, which has been reported as a "late" proinflammatory mediator in sepsis [2,3].

HMGB1 plays a role in many clinical conditions such as autoimmunity, acute ischemia-reperfusion injury, cardiovascular disease and cancer [4]. Recent evidences suggest that HMGB1 plays critical roles in the development and progression of numerous tumors [5]. HMGB1 modulates the transcriptional activity in the nucleus, but it is also present in the cytoplasm and outside the cell in certain conditions, associated with the proliferation and metastasis of many tumors, including breast cancer, colon carcinoma, and melanoma[6]. More recently, HMGB1 has been recognized as a proangiogenic factor [7].

In the case of tumors, HMGB1 recognition has a paradoxical dual effect: the reparative inflammatory response promotes tumor neoangiogenesis, cell survival, expansion, and metastases; on the other hand, it triggers protective anti-neoplastic T-cell responses[8,9]. Tumor cell death triggered by chemotherapy or radiotherapy initiates an immunoadjuvant pathway that contributes to the success of cytotoxic treatments. The interaction of HMGB1 released from dying tumor cells with Toll-like receptor 4 (TLR4) on dendritic cells (DCs) was required for the cross-presentation of tumor antigens and the promotion of tumor specific cytotoxic T-cell responses [10,11]. HMGB1 plays roles in various disease conditions mainly through RAGE (the receptor for advanced glycation end products). HMGB1-RAGE interactions have been found to be important in a number of cancers, which involves the MAPK/ERK pathway[12].

HMGB1 has emerged as a candidate for therapeutic intervention in various disease conditions [13]. However, further basic and clinical studies are warranted to confirm the roles played by HMGB1 in clinical cancer medicine. In the present study, the expression of HMGB1 protein was evaluated with tissue microarray(TMA) and immunohistochemical(IHC) staining procedures to study the prognostic significance of HMGB1 and its correlation with the clinical and histopathologic features of resectable gastric adenocarcinomas.

## Patients and methods

### Patients

TMAs were prepared for IHC test from a total of 78 consecutive cases of gastric adenocarcinomas operated in our department from December 2007 to October 2008. All the patients was given the radical resection and D1+or D2 lymphadenectomy followed by adjuvant chemotherapy with the regimen ECF (Epirubicin, cisplatin and 5-FU). To all patients, no preoperative therapy was given. The pathologic staging were made according to American Joint Committee on Cancer (AJCC) TNM staging system. The follow-up end point was defined as the recurrence or metastasis of the cancer. The use of the tissue samples in TMA analyses and clinical data was approved by Medical Ethics Committee of The Fourth Military Medical University and the patients. Patients' clinical and histopathologic data were summarized in Table 1.

**Table 1:** Clinical and histopathologic data of the patients.

Variables	Number of cases(%)
Number of patients	78(100%)
Age(y)	
≤ 60	44(56.4%)
> 60	34(43.6%)
Gender	
Male	55(70.5%)
Female	23(28.5%)
Tumor localisation	
Proximal	33(42.3%)
Distal	45(57.7%)
Histologic grade	
Undifferentiated(G4)	13(16.7%)
Poorly differentiated(G3)	27(34.6%)
Moderately differentiated(G2)	29(37.2%)
Well differentiated(G1)	9(11.5%)
Tumor stage	
Stage I + II	35(44.9%)
Stage III + IV	43(55.1%)
Primary tumor	
T1-2	12(15.4%)
T3-4	66(84.6%)
Regional lymph nodes	
N0	34(43.6%)
N1-3	44(56.4%)

### Tissue Microarrays

For each case, we selected the tumor foci for the TMA construction during routine diagnosis by marking them on the more representative hematoxylin-eosin (H & E)-stained slide with a waterproof pencil.

The advanced tissue arrayer (ATA-100, Chemicon International, Tamecula, CA, USA) was used to create holes in a recipient paraffin block and to acquire cylindrical core tissue biopsies with a diameter of 1 mm from the specific areas of the "donor" block. The tissue core biopsies were transferred to the recipient paraffin block at defined array positions. The TMAs contained tissue samples from 78 formalin-fixed paraffin-embedded cancer specimens with known diagnosis, and correlated non-cancerous tissues from the same patients.

The block was incubated in an oven at 45°C for 20 min to allow complete embedding of the grafted tissue cylinders in the paraffin of the recipient block, and then stored at 4°C until microtome sectioning.

### Immunohistochemical staining

Rabbit-derived anti-human HMGB1 antibodies were used for IHC detection of HMGB1 protein in TMAs. TMA sections were processed for IHC demonstration of HMGB1 protein by the Biotin-Avidin-Peroxidase detection system (Sigma). The anti-HMGB1 antibodies were used at 1:200 dilutions. Endogenous peroxidase was inhibited by incubation with freshly prepared 3% hydrogen peroxide with 0.1% sodium azide. Nonspecific staining was blocked with 0.5% casein and 5% normal goat serum. TMAs were incubated with biotinylated goat anti-rabbit antibodies and ExtrAvidin-conjugated horseradish peroxidase. Staining was developed with diaminobenzidine substrate and sections were counterstained with hematoxylin. Normal mouse serum or PBS replaced anti-HMGB1 antibodies in negative controls.

### The quantification evaluation of HMGB1 protein expression

HMGB1 expression was semiquantitatively estimated as the total HMGB1 immunostaining score, which was calculated as the sum of a proportion score and an intensity score. The propotion score reflects the fraction of positive staining cells(score 0, < 5%; score 1, 5% - 10%; score 2, 10 - 50%; score 3, 50 - 75%; score 4, > 75%). The intensity score represents the staining intensity(score 0, no staining signal; score 1, weak positive signal; score 2, moderate positive signal; score 3, strong positive signal). Finally, a total expression score was given ranging from 0 to 12. Based the analysis in advance, the overexpression of HMGB1 was defined as the total expression score ≥ 9.

### Statistical analysis

Results are expressed as median and range. For statistical analysis, the Chi-square test was made with the software GraphPad Prism, and uni-and multivariate analysis and survival analysis were made with the SPSS 16.0. Significance was defined as *P *< 0.05.

## Results

### The expression of HMGB1 protein in the gastric adenocarcinomas

**Figure 1:**
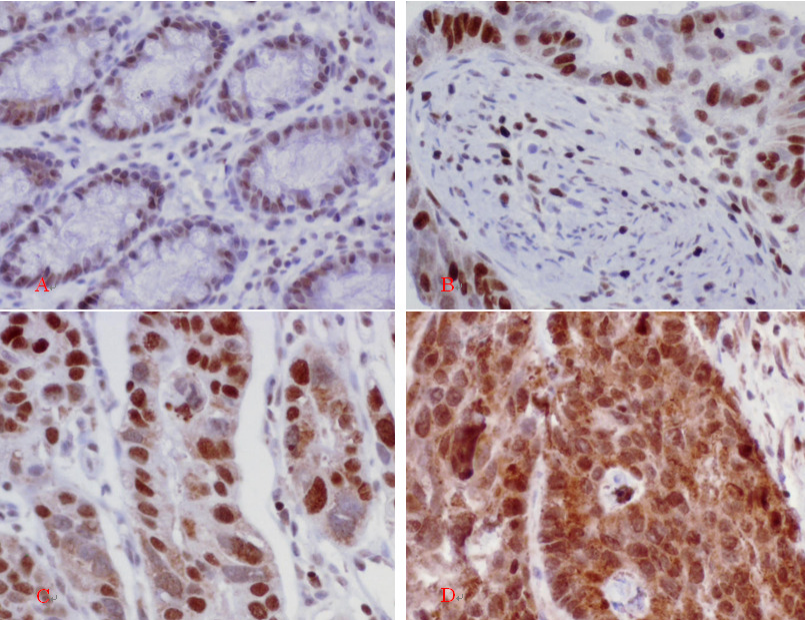
**Immunohistochemical detection of HMGB1 protein in different gastric tissues**. A: Normal rectal sample. The low-expression of HMGB1 was detected in epithelial and stromal cells B: Gastric adenocarcinoma sample with well differentiation. The immunohistochemical staining showed strong positive signal (+ + +) in the cancer cells, and low-expression was detected in the stromal cells, which localized in the nucleus. C: Gastric adenocarcinoma sample with poor-moderate differentiation. The immunohistochemical staining showed strong positive signal (+ + +), which mainly localized in the nucleus. D: In rare cases, the strong staining was detected in the nucleus and cytoplasm of the cancer cells. Original magnification × 200.

Expression of HMGB1 protein was evaluated by using immunohistochemical staining. As a nonhistone DNA-binding protein, the expression of HMGB1 protein was mainly localized in the nucleus. In gastric adenocarcinoma cells, the expression of HMGB1 protein was also mainly detected in the nucleus (Figure 1B, C). But in rare cases of sample, the positive staining could be found in nucleus and cytoplasm (Figure 1D).

The positive staining was detected in most of gastric adenocarcinoma cells. HMGB1 unexpressed tumors mainly were found in the poorly differentiated adenocarcimas. The difference of HMGB1 expression in peritumoral and normal (distant) tissues was not assessed based on the histopathologic changes and HP status. The positive staining was detected in 69/78(88.5%) adenocarcinoma cells, and 61/78(78.2%) in non-cancerous cells with no significant difference (*P *= 0.202, Table 2). But the rate of HMGB1 overexpression (total expression score ≥ 9) was elevated in gastric adenocarcinoma cells, compared with corresponding non-cancerous cells (41.0% *vs*. 9.0%, *P *< 0.001).

**Table 2:** Expression of HMGB1 in cancerous tissues and correlated non-cancerous tissues.

Variables	All cases(n)	Positive expression		Significance	Overexpression		Significance
		(n=)	(%)	(*P*)	(n=)	(%)	(*P*)
Cancer	78	69	88.5	0.202	32	41.0	< 0.001
Non-cancer	78	61	78.2		7	9.0	

### The correlation of HMGB1 protein expression with the clinical and histopathologic characteristics

The relationship between HMGB1 overexpression and various clinical and histopathologic features was analyzed. No significant correlation was found between HMGB1 overexpression with age, or gender (Table 3). As shown in Table 3, the statistically significant difference was found in the groups with district differentiation (*P *= 0.012). But, except the significantly elevated rate in G1 group, the difference was no found in G2, G3, and G4 group, compared with the other two groups. The phenomenon perhaps was induced by the distribution bias of the available cases.

**Table 3:** Expression of HMGB1 in correlation with clinicopathologic variables.

Variables	cases(n)	HMGB1 low < 9		HMGB1 high(≥ 9)		**Significance(*P*)**
		(n=)	(%)	(n=)	(%)	
Total	78	46	59.0	32	41.0	
Gender						
Male	55	29	52.7	26	42.3	0.129
Female	23	17	73.9	6	26.1	
Age at surgery						
≤ 60	44	29	65.9	15	34.1	0.111
> 60	34	16	47.1	18	52.9	
Tumor differentiation						
G1	9	1	11.1	8	88.9	0.012
G2	29	21	72.4	8	27.6	
G3	27	17	67.0	10	37.0	
G4	13	7	53.8	6	46.2	
Tumor stage						
Stage I + II	35	23	65.7	12	34.3	0.356
Stage III + IV	43	23	53.5	20	46.5	
pT stage						
pT1-2	12	9	75.0	3	25.0	0.340
pT3-4	66	37	56.1	29	43.9	
Nodal status						
pN0	34	23	67.6	11	32.4	0.246
pN1~3	44	23	52.3	21	47.7	

According to the pathologic TNM staging, the cases were divided into two groups: stage I + II and stage II + IV. The group with early stage showed elevated rate of HMGB1 over-expression, but no statistically significant difference was found between the two groups (34.3% vs. 46.5%, *P *= 0.356). Then, the cases were divided into two groups with lymph node metastasis or no. The rate of HMGB1 overexpression was 21/23(47.7%) in cancerous specimens with lymph node metastasis, compared with 11/23(32.4%) in cancerous specimens without lymph node metastasis. But no significant difference was found (*P *= 0.246). While, primary tumor infiltrating depth perhaps correlated with HMGB1 overexpression. pT3 + 4 group showed elevated rate of HMGB1 overexpression compared with pT1 + 2 group, but no statistical difference was found (43.9% *vs*. 25.0%, *P *= 0.340) (Table 3).

### Kaplan-Meier survival analysis

Regarding the results of cancer-free survival analysis, there was no correlation between gender, age, location, grade of the tumor with prognosis. But the tumor stage and HMGB1 overexpression showed the correlation with cancer-free survival. Survival curves were plotted according to the Kaplan-Meier method for the patients with HMGB1 expression status and stage. Tumor stage had a significant effect on cancer-free survival for stage I+II tumors compared with stage III + IV tumors(*P *= 0.022). The expected survival time was 19.0000 ± 7.35247 m for Stage I + II tumors (95% CI = 16.4743 - 21.5257), 16.4186 ± 8.69108 m for stage III + IV tumors (95% CI = 13.7439 - 19.0933).

**Figure 2:**
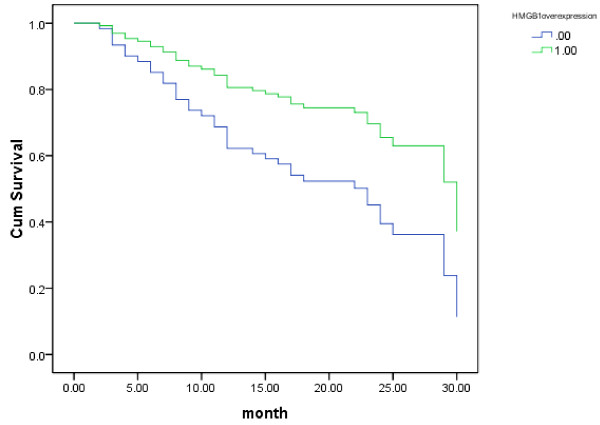
**Kaplan-Meier curves of cancer-free survival for HMGB1 overexpression-positive(1) and -negative(0) gastric cancer cases**.

Furthermore, survival analysis revealed that HMGB1 overexpression affected cancer-free survival. There was significant difference in cancer-free survival between groups with HMGB1 overexpression and with its low-level expression (*P *= 0.023, Figure 2). Multivariate analysis showed that the expected cancer-free survival time was 20.4375 ± 7.28648 m for tumors with HMGB1 overexpression (95% CI = 17.8104 - 23.0646), 15.5870 ± 8.23158 m for tumors with HMGB1 no-and low-level expression (95% CI = 13.1425 - 18.0314). HMGB1 overexpression was an independent predictor of cancer-free survival for patients with resectable gastric adenocarcinomas. Furthermore, we analyzed the characteristics of the patients with HMGB1 overexpression who died during the follow-up period. We found the most of the cases had a relatively late disease (Table 4).

**Table 4:** The clinical and histopathologic characteristics of the patients with HMGB1 overexpression who died during the follow-up period.

No.	Gender	Age	Survival Time(m)	pTNM Stage	Grade
1	male	75	15	T4N1M1	G1
2	male	69	23	T3N1M0	G2
3	female	48	12	T3N1M0	G3
4	male	66	12	T3N3M0	G3
5	male	65	11	T3N3M0	G3
6	female	51	9	T4N1M0	G4
7	male	61	10	T4N1M0	G4
8	female	58	7	T3N2M0	G2
9	male	68	8	T3N2M0	G2
10	male	52	11	T3N2M0	G4
11	male	69	10	T4N1M0	G3
12	female	75	14	T4N2M0	G4

## Discussion

The occurrence and development of GC correlated with various molecular and genetic incidents. To investigate the significance of the molecular expression in GC may help us to identify potential treatment target and(or) predictive marker of prognosis and treatment response. Overexpression as well as cytoplasmic localization of HMGB1, particularly in conjunction with its receptor for advanced glycation end products (AGEs), is associated with the proliferation and metastasis of many tumor types [14-16]. Furthermore, HMGB1 secreted from primary tumors decreased the number of macrophages to attenuate the anti-metastatic defense in patients with colorectal cancers, through inducing growth inhibition and apoptosis in macrophages[17,18]. HMGB1 can also influence a variety of important cell types within the tumor microenvironment, including fibroblasts, leukocytes, and vascular cells[19]. So, targeting the HMGB1 ligand or its receptor represents an important potential application in cancer therapeutics [20].

But, HMGB1 may play a controversial role in the occurrence and progression of cancers. Riuzzi F *et al*. reported that the HMGB1-RB interaction perhaps induced the HMGB1-mediated transcriptional repression, cell growth inhibition, G1 cell cycle arrest, apoptosis induction, and tumor growth suppression[21]. Furthermore, the functional inactivation of RAGE in myoblasts results in reduced myogenesis, increased proliferation, and tumor formation *in vivo *[22]. On the other hand, the tumor cell death triggered by chemotherapy or radiotherapy initiates an immunoadjuvant pathway that contributes to the success of cytotoxic treatments. After DNA-alkylating damage, the activation of PARP regulates the translocation of HMGB1 from the nucleus to the cytosol[23]. The interaction of HMGB1 protein released from dying tumor cells with TLR4 on DCs was required for the cross-presentation of tumor antigens and the promotion of tumor specific cytotoxic T-cell responses[10,11,24], which are selectively involved in the cross-priming of anti-tumor T lymphocytes *in vivo *[25,26]. The controversy indicates that HMGB1 may affect the treatment response of cancers, and HMGB1 may affect the prognosis through complicated pathways.

Of course, the main stream of the study on HMGB1 is that it has the positive correlation with the occurrence, progression, and metastasis of cancers. HMGB1 expressed and secreted by cancer cells are associated with increased metastasis and poorer outcomes in a wide variety of tumors. HMGB1 levels are related with the clinicopathologic characteristics in many cancers. Cheng et al. reported the serum HMGB1 protein levels in hepatocellular carcinoma was significantly higher than those in chronic hepatitis, liver cirrhosis and healthy control, and positive correlation were found between HMGB1 and alpha-fetoprotein, and between HMGB1 and the size of tumor. HMGB1 were significant differences among Edmondson grade, TNM stage and Cancer of the Liver Italian Program score[27]. The similar results were also obtained in the study on GC [28].

The study on the correlation of between HMGB1 expression and gastrointestinal cancers can be found recently. Akaike et al. reported the expression of HMGB1 in GC cells with the intestinal type was significantly increased compared to that in the diffuse type, which was positively correlated with the degree of macrophage infiltration inside the tumor microenvironment. And the prognosis of the low HMGB1 group was significantly poorer than that of the high HMGB1 group [29]. Völp *et al*. reported HMGB1 gene was overrepresented in one third of colon cancers. Correspondingly, HMGB1 protein levels were significantly elevated in 90% of the 60 colon carcinomas tested compared with corresponding normal tissues evaluable from the same patients [30]. HMGB1 overexpression was significantly associated with tumor invasion, lymph node metastasis, distant metastasis and Duke's stage, and inversely associated with overall survival [31].

In the present study, the expression of HMGB1 was detected in most of the gastric adenocarcinoma samples, as well as the borderline and normal epithelial cells. But the increased expression of HMGB1 protein was found in cancer samples, compared with the borderline and normal (distant) tissues. As we have found, the positive staining signals mainly detected in nucleus of gastric adenocarcinoma cells and stromal cells of cancerous tissues. In rare cases, the strong staining was detected in the nucleus and cytoplasm of the cancer cells. In our another study, there was a higher rate of cytoplasm staining in colorectal cancer cells(data not shown here). The mechanism and the significance need further study.

In the study, the rate of HMGB1 overexpression tended to increase correlated with invasion depth, tumor stage, and lymph node. But no statistical difference was found, which had acceptable difference with the currently reported results. It perhaps indicates that more sensitive and stable methods are needed for the further study. But it was confirmed that gastric adenocarcinoma showed a high rate of HMGB1 overexpression (total expression score ≥ 9). In the group of patients, 32/78(41.0%) showed the overexpression of HMGB1.

Tumor stage is the current marker of prognosis of GC. In the group of patients, survival analysis showed that tumor stage inversely correlated with cancer-free survival. Furthermore, the survival analysis showed that HMGB1 overexpression positively associated with the cancer-free survival of patients with resectable gastric adenocarcinoma. For GC patients with HMGB1 overexpression, they might have more chance to have a long recurrence-free survival time after curative resection followed adjuvant chemotherapy with ECF regimen.

In conclusion, the high-level expression of HMGB1 protein was detected in gastric adenocarcinoma cells. It consisted with the other researchers' reports. In many gastric adenocarcinomas, the overexpression of HMGB1 was found. The overexpression of HMGB1 was positively correlated with the prognosis of the patients given curative resection and adjuvant chemotherapy.

## Competing interests

The authors declare that they have no competing interests.

## Authors' contributions

GB supervised research project, participated in the data collection, drafted the manuscript. QQ participated in the data collection, supervised ICH. HZ carried out the operation. XH carried out the operation, acted as corresponding author and did the revisions. All authors read and approved the final manuscript.
